# Parental Attitudes, Behaviors, and Barriers to School Readiness among Parents of Low-Income Latino Children

**DOI:** 10.3390/ijerph15020188

**Published:** 2018-01-24

**Authors:** Jaime Peterson, Janine Bruce, Neel Patel, Lisa J. Chamberlain

**Affiliations:** 1 Department of Pediatrics, Stanford School of Medicine, Palo Alto, CA 94305, USA; jsbruce@stanford.edu (J.B.); lchamberlain@stanford.edu (L.J.C.); 2 Fair Oaks Health Center, San Mateo Medical Center—San Mateo County Health System, San Mateo, CA 94063, USA; patelnd@pamf.org

**Keywords:** school readiness, pediatrics, low-income children, parents

## Abstract

We sought to explore parental attitudes, behaviors, and barriers regarding school readiness in a county clinic serving low income, Latino children. Between December 2013–September 2014, we conducted a cross sectional survey of parents during 3–6 years well-child appointments about school readiness (SR) across: (1) attitudes/behaviors; (2) barriers; and (3) awareness; and (4) use of local resources. Most parents (n = 210, response rate 95.6%) find it very important/important for their child to know specific skills prior to school: take turns and share (98.5%), use a pencil and count (97.6%), know letters (99.1%), colors (97.1%), and shapes (96.1%). Over 80% of parents find education important and engage in positive SR behaviors: singing, practicing letters, or reading. Major barriers to SR were lack of knowledge for kindergarten readiness, language barriers, access to books at home, constraints on nightly reading, difficulty completing school forms, and limited free time with child. Awareness of local resources such as preschool programs was higher than actual utilization. These low-income, Latino parents value SR but lack knowledge to prepare their child for school and underutilize community resources such as free preschool programs. Pediatricians are uniquely positioned to address these needs, but more evidence-based interventions are needed.

## 1. Introduction

Children who enter school ready for kindergarten are more likely to succeed academically [1,2,3,4,5]. The metric of “School Readiness” (SR) measures a child’s level of being ready for kindergarten across five domains: cognitive development, physical development, language development, self-help skills, and social–emotional development [6]. Children who enter school behind their peers are less likely to catch up by graduation, are more likely to drop out of school, experience teen pregnancy, or be incarcerated as juveniles [1,3,4,7].

Society promotes school readiness [8,9,10] through the health and education systems. First, the child health system promotes physical and language development with near universal health coverage of children and pregnant women [11], as healthy children do better in school than children with acute or chronic disease [7,12]. The health system also serves as a referral source, linking parents to programs such as the Nurse–Family Partnership, Early Intervention, and the Special Supplemental Nutrition Program for Women, Infants, and Children [13,14,15]. Clinic based SR interventions are limited [16,17,18,19], but have successfully contributed to increased school readiness behaviors, such as book sharing and daily reading, among low-income children. Outside the child health system, society invests in early childhood education programs such as Head Start programs [4,9]. High quality, early child education programs have a demonstrated positive return on the investment [2,20] and support early cognitive, language, and self-help skill development for SR [1,2,21]. 

Despite these societal investments the United States (US) lacks a comprehensive early childhood system. The US lags behind 28 high-income countries in preschool enrollment with only 54% of 3 and 4 year-olds in preschool, the majority of whom are from upper income families [22,23]. Nearly 100% of low-income and minority children access routine health care at community clinics, yet there is no universal access to early education. While over one million low-income children attend Head Start annually [9], the most at risk children often do no enroll in preschool [4,5,22,23,24], struggle to access community resources [23,25], and ultimately, 80% will enter kindergarten behind their more advantaged peers [3,4,5,10,23].

Those at highest risk for poor school readiness are children living in poverty, “English language learners” (ELL) and those with mothers with low maternal education levels [3,4,5,6,23]. In the US, Latinos are a growing segment of the population with the largest Latino population residing in California. The state is home to over 14 million Latinos, 4.6 million of whom are children [26], 51% of the total child population. Among the Latino child population in California, 22% are ELL [4,23]. This growing segment of children has unique SR needs and there is a paucity of evidence on how to best support their SR [4,23]. Latinos have robust birth outcomes, overall good physical health, and strong social–emotional readiness but cognitive gaps are notable as early as 24 months and persist at school entry [27,28]. There is a need for more coordination in the critical early years to improve SR, however barriers of cost and inadequate number of subsidized spots exist [4,9,25]. High quality preschool programs can close this cognitive gap [1,2,20,21]. However, Latino children have the lowest preschool participation rate of any ethnicity in the US [4]. Until universal access to high quality preschool exists, parents are unaided as their child’s ‘first teacher’, thus we need to better understand how low-income Latinos parents perceive and value SR, as prior studies are few and were primarily amongst white and African American families [8,23,29]. These findings are the critical first step in creating a clinic-based intervention to directly support SR for all children, but particularly for Latino children. 

Community based pediatricians are uniquely positioned to improve school readiness given their unparalleled access to children who are at risk for not being ready for kindergarten [10,30]. Pediatricians also enjoy high levels of family trust, enabling them to support these families between ages 0–5. They have the opportunity to engage parents in SR and bridge the divide between the health and education systems to ensure all children are school ready [10]. Given the unique SR needs of low-income, Latino families and the relatively limited data on their perceived SR needs and barriers, we sought to understand how low-income, Latino parental attitudes, behaviors, and barriers in order to support their young children’s school readiness in a community based, pediatric clinic. 

## 2. Materials and Methods 

### 2.1. Study Setting and Participants

We conducted a cross sectional survey of all ‘parents or guardians’ (parents) of children who had a well-child check in high-density suburban county operated health clinic that serves both adults and children in Northern California between 4 December 2013 and 31 September 2014. The clinic is the medical home for approximately 900 children 4–6 years old. The pediatric clinic’s patients are 94% Hispanic, 2% white, and 1% black. In 2012, the preferred language was Spanish (85%) with 73% of patients requiring a bilingual provider. Eighty-seven percent of children rely upon Medicaid or the Children’s Health Insurance Program. Individual parent and child demographics were not collected to promote a sense of anonymity, as demographic data was available at the level of the clinic. The Human Subjects Committee of the Institutional Review Boards of Stanford University (IRB e-protocol #28860) and San Mateo Medical Center approved and oversaw the conduct of this study. Participants reviewed a waiver of documentation of consent before completing the survey. 

### 2.2. Eligibility Criteria

All parents of children between three and six years of age attending a well-child check between 4 December 2013 and 31 September 2014 were asked by a bilingual ‘patient service associate’ (PSA) to complete the SR survey before seeing a pediatric provider for their scheduled appointment. Parents were excluded if their child was attending an acute visit or was less than three years of age or older than six years of age, regardless of other children three to six years of age in the home. 

### 2.3. Survey Instrument Development

A 56-item self-administered questionnaire was adapted from the 2007 School Readiness Parent Survey portion of the US Department of Education National Household Education Surveys Program [31]. The adapted survey assessed school readiness across four domains: parental attitudes and behaviors; barriers; and awareness and use of local resources. The original NHESP does not include items regarding SR barriers, thus, 10-items were created based on SR barriers in the literature [6,7]. The SR resources items were modified to include the names of local resources to better explore local utilization patterns. Likert scales assessed parental attitudes regarding key SR skills (1 = not important, 2 = important, 3 = very important) and parental attitudes regarding school readiness and barriers to school readiness (1 = strongly disagree/disagree, 2 = neutral, 3 = agree/strongly agree). Parent behaviors were assessed based on a ‘yes’ or ‘no’ response for a behavior within the last week. Parents were asked if they (1) were aware of 10 different local community SR resources, and (2) if they had used any of them in the last year. The resources included local school services, community agencies (parent preschool groups or parenting classes), the local library, and non-profit, state and federal preschool programs. The survey was reviewed by content experts from Developmental and Behavioral Pediatrics, translated into Spanish, pilot tested among providers (n = 10) and parents (n = 28), and repeatedly refined to ensure validity and cultural relevance in both English and Spanish prior to study initiation. 

### 2.4. Statistical Analysis

Statistical analyses were performed using SPSS statistical software, version 22 (IBM, Armonk, NY, USA). We calculated descriptive frequencies on all variables in each survey domain as counts with percentages for each variable. 

## 3. Results

A total of 229 parents participated with an overall response rate of 95.6%. Nineteen respondents did not complete at least 50% of the survey and those surveys were excluded, leaving a final sample of 210 parents for analysis. 

### 3.1. Parent Attitudes and Behaviors around SR

Parent attitudes regarding their preschool child’s school readiness showed that parents overwhelmingly agreed/strongly agreed their child should have specific skills before entering school. The majority of parents viewed it to be very important/important for their child to be able to take turns and share (98.5%); use a pencil and count (97.6%); and know letters (99.1%), colors (97.1%), and shapes (96.1%). The majority of parents reported positive literacy behaviors in the last week. More than 80% of parents reported singing, practicing letters, telling a story or reading to their child daily in the last week. Over half (55%) of parents reported visiting the library with their child in the last week (Table 1). 

### 3.2. Parent Barriers to SR

Parent barriers to promoting school readiness for their preschool children included parents not knowing what their child needs to know for kindergarten (34% strongly agree/agree) language barriers (31.4% strongly agree/agree); limited access to books in the home (24.1% strongly agree/agree); time constraints on nightly reading (23.7% strongly agree/agree); difficulty completing school forms (19.9% strongly agree/agree); and limited free time with child due to employment (19.6% strongly agree/agree). Parents did not report that reading, transportation, care of other children or other responsibilities were barriers to support their child’s school readiness (68–75% of parents strongly disagreed/disagreed) (Table 2). 

### 3.3. Awareness and Utilization of SR Resources

**Figure 1:**
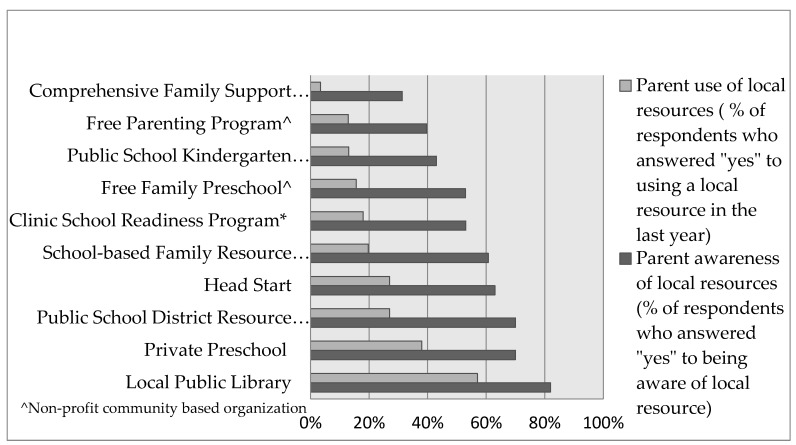
Parent awareness and use of local school readiness services in the last year (n = 210). * A unique service offered by clinic.

There was a gap between parental awareness and use of local school readiness resources in the last year. Parents were generally aware of a school-based family resource center, Head Start, public school district resource center, private preschools, and local libraries. However, utilization rates of these resources ranged between 20–50% in the last year. For example, despite 63% awareness of Head Start, only 27% used a Head Start program in the last year. Conversely, there was generally less than 50% awareness and very low utilization (<20% used in the last year) of family support programs, parenting groups, kindergarten prep courses, free family preschools, and the clinic based school readiness program. Despite resource utilization lagging behind resource awareness, the SR resources with higher awareness levels tended to have more utilization (Figure 1). 

## 4. Discussion

Parents of low-income Latino children value school readiness and feel responsible for preparing their child for kindergarten. Parents did not report that learning begins with school entry, a commonly held immigrant belief [25]. Additionally, parents correctly identified some key skills their children need before entering kindergarten (colors, counting, letters, sharing, etc.). This supports assertions that while low-income Latino children often enter kindergarten behind their peers, parents care about school preparation and want to provide opportunities to support early skill development [32]. 

There appears to be a gap between Latino parent attitudes and behaviors and their use of community resources to develop these SR skills. While parents value school readiness, see their role as important and can recognize important skills, the highest reported barrier to SR was lack of parent knowledge of what their child needs to know for kindergarten. Parents report positive early literacy behaviors with their child like library visits, reading, singing, and practicing letters in the last week. Interestingly, parents were often aware of the community resources, while the utilization was quite low (<30% used in the last year) with exception of the library. The literature suggests that a combination of structural, informational/bureaucratic, and cultural barriers contribute to accessibility of early childhood education resources [25]. There are many plausible reasons for the underutilization in this Latino study population: waitlists, time constraints (e.g., low-income parents often work multiple jobs), transportation (e.g., lack of public transportation options), burdensome documentation, misinformation (e.g., rumors that personal information will be shared with authorities), reputation of community programs (e.g., poor customer service), fear of immigration enforcement and cultural values (e.g., many Latino families opt out of preschool due to a preference for the child to be at home until age 3). While the direct consequences of underutilization were not explored in this study, it is not unreasonable to consider underutilization as a benchmark for a child’s general SR environment: lower utilization may result in less reading, lower preschool enrollment, and overall lower SR [25]. There is a need to further explore how these factors contribute to the low resource utilization and the direct impact on a child’s SR. 

The United States lacks a coherent early child education system. The combination of high trust [8,33] and near universal access to 0–5 years old give pediatricians an important opportunity to address gaps in early childhood education, particularly for the most disenfranchised communities. Despite this opportunity, pediatricians struggle to support early school readiness for parents living in poverty due to various factors including limited evidence-based interventions. The current standard of care is Reach Out And Read (ROAR), a national program where pediatricians provide books and model developmentally appropriate reading practices to low-income patients [6,16]. More interventions are emerging and supported within the American Academy of Pediatrics and the Academic Pediatrics Association strategic, multiyear focus to ameliorate poverty [34]. Several examples include Puerto Rican parents who were taught a drill to practice letters with their child which led to significant improvement, regardless of the parent’s education or English proficiency [35]. A second intervention, the Video Interaction Project, improved infant stimulation and parent reading after guided parent–child interactions in clinic [19]. A third improved Head Start enrollment when a clinic based referral system was implemented [36]. The challenge of scalability of the latter intervention is the assumption that sufficient Head Start spots exist. Finally, because parents report libraries as the most frequented community resource, library card distribution via clinic “prescriptions” are in early stages of exploration [37]. Continuing to build the evidence for clinic based, culturally tailored, and feasible early childhood educational interventions is an important step to improve school readiness rates in low-income communities. 

While the evidence emerges for clinic based, SR interventions, pediatricians can continue to encourage preschool enrollment. There is much to learn from the early childhood programming already in place, such as the role family engagement plays in state and federal preschool programs (like Head Start) to support parents on how to best prepare their child for school. Additionally, pediatricians and child health systems can advocate for the expansion of subsidized preschool spots at the local and state level as many states lack sufficient preschool spots for eligible children [5,25]. 

The present study has several limitations. We surveyed a relatively small sample of Latino parents from a single community pediatrics clinic in Northern California, and thus, our findings are generalizable only to clinics serving similar demographics (low-income, Latino, and primarily Spanish speaking families). Since we only surveyed parents who attended a well-child check, there is a selection bias for parents that are able to attend appointments. As such, our findings are likely conservative, as parents who miss appointments are likely more vulnerable and face more barriers to SR. Finally, individual demographic information was not collected, to ensure privacy and encourage participation. We surmise that demographic data at the clinical level represents an accurate representation of this homogenous, county-based pediatric clinic, with a Healthcare Provider Shortage Area score of 6. Future analysis with both qualitative methods and individual level demographic information of a larger study size will allow for parent sub-group identification for those facing the most risk factors, those with more resilience and better understanding of utilization barriers. 

## 5. Conclusions

To date, recommendations are limited for pediatricians to address children at high risk for poor school readiness or how to implement targeted school readiness interventions beyond Reach out and Read. This study suggests that low-income, Latino parents value SR and practice positive literacy behaviors yet do not know what their child needs to know at kindergarten entry and underutilize key SR resources in the community. A deeper understanding is needed to support parent modeling and connecting to local SR resources to help them better prepare their child for school. As a trusted community resource with access to otherwise disconnected families, pediatricians are well positioned to bridge this gap. For the child health system to support what has traditionally been within the purview of the education system more evidenced-based clinical interventions are needed. 

## Figures and Tables

**Table 1:** Parent attitudes and behaviors regarding school readiness of their preschool aged children (n = 210).

**Domain 1: Parent Attitudes Regarding School Readiness Skills**	Percent % *		
How important is it for a child to be able to do the following before they start kindergarten:	Very Important	Important	Not Important
Take turns and share	67.3	31.2	1.5
Use a pencil	66.8	30.8	2.4
Know letters of the alphabet	64.6	34.5	1.0
Count to 20	66.8	30.8	2.4
Recognize 5 basic colors	59.5	37.6	1.4
Recognize 5 basic shapes	58.7	37.4	3.9
**Domain 2: Parent Attitudes Regarding Their Role in School Readiness**			
Please indicate level of agreement with the following statements about preparing your child for kindergarten:	Strongly Agree or Agree	Neutral	Disagree or Strongly Disagree
It is important to me and my family	94.3	2.4	3.3
It will help my child succeed later in school	93.3	2.9	3.8
It is my responsibility as a parent	89.3	4.4	6.3
It is the responsibility of the school	41.0	24.5	34.5
**Domain 3: Parent Behaviors to Promote School Readiness**			
In the last week, did anyone do the following with your child:	Yes		
Teach your child songs or music	91.8		
Teach your child the letters of the alphabet	87.1		
Tell your child a story	87.1		
Read to your child every day	81.6		
Do arts and crafts with your child	75.5		
Visit a library with your child	55.2		

* Percentages in rows and columns may not sum to 100% because of rounding.

**Table 2:** Parent barriers to school readiness (n = 210).

	Percent % *		
*State your level of agreement with the following statements about why it can be difficult to prepare your child for school.*	Strongly Agree or Agree	Neutral	Disagree or Strongly Disagree
I do not know what my child needs to know for kindergarten	34	23.9	42
English is difficult for me	31.4	15.5	53.1
We do not have books in our home	24.1	11.3	64.6
We do not have time to read every night before bed	23.7	10.3	66
I do not know how to complete school registration forms	19.9	14.3	65.8
My job makes it difficult to spend time with my child	19.6	12.4	68
Reading is hard for me	18.1	9.8	72
Transportation to places like the library is difficult	16.8	14.7	68.4
I have to take care of other children	12.6	12	68.6
Other responsibilities take priority	12.5	12.5	75

* Percentages in columns may not sum to 100% because of rounding.
